# Examining clinical similarities between myalgic encephalomyelitis/chronic fatigue syndrome and d-lactic acidosis: a systematic review

**DOI:** 10.1186/s12967-017-1229-1

**Published:** 2017-06-07

**Authors:** Amy Wallis, Michelle Ball, Sandra McKechnie, Henry Butt, Donald P. Lewis, Dorothy Bruck

**Affiliations:** 1 Psychology Department, College of Health and Biomedicine, Victoria University, PO Box 14428, Melbourne, VIC 8001 Australia; 2 College of Engineering & Science, Victoria University, Melbourne, VIC Australia; 3 Bioscreen Yarraville (Aust) Pty Ltd, Melbourne, VIC Australia; 4 CFS Discovery Clinic, Melbourne, VIC Australia

**Keywords:** Acidosis, lactic, Dysbiosis, Fatigue syndrome, chronic, Encephalomyelitis, myalgic, Microbiota-gut–brain, Neurological symptoms

## Abstract

**Background:**

The pursuit for clarity in diagnostic and treatment pathways for the complex, chronic condition of myalgic encephalomyelitis/chronic fatigue syndrome (ME/CFS) continues. This systematic review raises a novel question to explore possible overlapping aetiology in two distinct conditions. Similar neurocognitive symptoms and evidence of d-lactate producing bacteria in ME/CFS raise questions about shared mechanisms with the acute condition of d-lactic acidosis (d-la).

**Methods:**

d-la case reports published between 1965 and March 2016 were reviewed for episodes describing both neurological symptoms and high d-lactate levels. Fifty-nine d-la episodes were included in the qualitative synthesis comparing d-la symptoms with ME/CFS diagnostic criteria. A narrative review of d-la mechanisms and relevance for ME/CFS was provided.

**Results:**

The majority of neurological disturbances reported in d-la episodes overlapped with ME/CFS symptoms. Of these, the most frequently reported d-la symptoms were motor disturbances that appear more prominent during severe presentations of ME/CFS. Both patient groups shared a history of gastrointestinal abnormalities and evidence of bacterial dysbiosis, although only preliminary evidence supported the role of lactate-producing bacteria in ME/CFS.

**Limitations:**

Interpretation of results are constrained by both the breadth of symptoms included in ME/CFS diagnostic criteria and the conservative methodology used for d-la symptom classification. Several pathophysiological mechanisms in ME/CFS were not examined.

**Conclusions:**

Shared symptomatology and underlying microbiota–gut–brain interactions raise the possibility of a continuum of acute (d-la) versus chronic (ME/CFS) presentations related to d-lactate absorption. Measurement of d-lactate in ME/CFS is needed to effectively evaluate whether subclinical d-lactate levels affect neurological symptoms in this clinical population.

**Electronic supplementary material:**

The online version of this article (doi:10.1186/s12967-017-1229-1) contains supplementary material, which is available to authorized users.

## Background

Myalgic encephalomyelitis/chronic fatigue syndrome (ME/CFS) is a complex condition with evidence of multi-systemic dysfunction. The primary symptom of post-exertional fatigue is accompanied by heterogeneous neurological, immune, cardiovascular, respiratory and/or gastrointestinal manifestations [1]. Research efforts continue to search for biomarkers to aid etiological understandings and treatment options for this debilitating condition. It has been proposed that some neurological symptoms may be related to an imbalance of commensal gut bacteria (i.e., gut dysbiosis [2]). Within ME/CFS, evidence of gut dysbiosis [3, 4] and associations between microbial genus and symptom expression [5] raise questions about whether gut dysbiosis plays a causative or mechanistic role in onset, maintenance and/or symptomatic variability. The mechanisms are not clear because microbe–gut–brain interactions can occur through several pathways (i.e., central, autonomic, and enteric nervous systems; neuroendocrine and neuroimmune; enteric microbiota) [6–8]. Investigations of other conditions with similar presentations may aid the current etiological understanding of ME/CFS. d-Lactic acidosis (d-la) is an acute condition that shares some similar features with ME/CFS and provides a clear example of the microbe–gut–brain interaction.

d-la is a type of metabolic acidosis with the primary presentation of encephalopathy (i.e., impaired mental state including confusion, loss of memory or cognitive capacity) [9]. d-la has also been referred to as “d-lactate neuropathy” or “d-lactate encephalomyelitis” in humans and “floppy kid syndrome”, or “drunken lamb syndrome” in animals. Originally described in ruminants [10], the condition has now been observed in multiple human case reports since 1979 [11].

The neurological symptoms and associated biochemical imbalances of d-la appear to result from gastrointestinal dysfunction. d-la is most commonly observed in patients with short bowel syndrome (SBS), often after surgery or removal of a section of the small bowel [12]. This reduced length diminishes the small bowel’s functional capacity to effectively metabolise carbohydrates leading to excessive bacterial fermentation in the colon [13]. Small intestinal carbohydrate malabsorption precipitates an increase in colonic acidity and the consequential overgrowth of certain species of colonic microbiota that produce an abundance of d-lactate. Healthy humans have the capacity to effectively metabolise d-lactate [14, 15]. However, the combination of high levels and insufficient d-lactate metabolic capacity can result in excess accumulation of d-lactate in the blood and absorption within the brain, resulting in the neurological symptoms characteristic of d-la [13].

Higher levels of d-lactate producing bacteria (such as *Streptococcus* and *Enterococcus*) have been identified in stool samples from patients with ME/CFS [4]. This evidence, combined with some similar neurological symptoms in both conditions, has led to comparison with d-la and proposal of the d-lactate hypothesis for ME/CFS. Accordingly, this hypothesis suggests that an increased abundance of d-lactate producing bacteria and suspected higher circulating levels of d-lactate may contribute to the neurological manifestations of ME/CFS. However, this hypothesis has not been systematically evaluated. Neither plasma nor urine d-lactate levels have been documented in ME/CFS to date. This lack of clinical d-lactate data coupled with confusion surrounding the degree of symptom overlap between d-la and ME/CFS provide the rationale for this qualitative review. To help ascertain the relevance of the d-lactate hypothesis for ME/CFS, Part A of this review aims to (a) systematically summarise published d-la episodes that report neurological symptoms and d-lactate levels; and (b) examine the overlap between d-la and ME/CFS symptom. Part B provides a narrative review of proposed neurological mechanisms in d-la to examine its relevance for ME/CFS aetiology.

## Main text

### Part A. Systematic qualitative review

#### Method

**Fig. 1:**
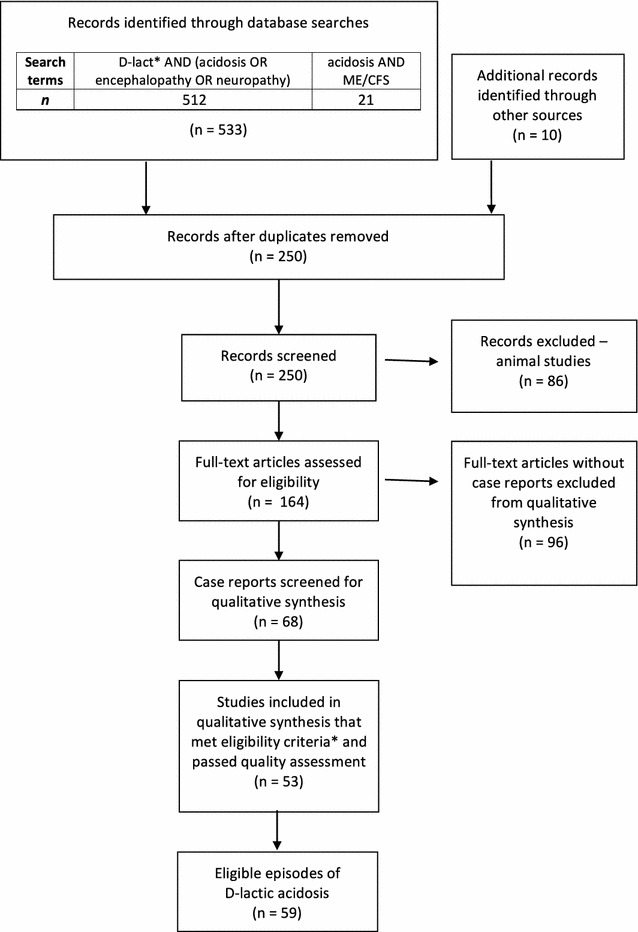
PRISMA flowchart of systematic search and article selection. *Asterisk* eligibility criteria included episodes of d-lactic acidosis where both neurological symptoms and d-lactate levels were reported

MEDLINE (via Ebscohost) and PubMed databases were searched for publications from 1965 to April 1 2016. To obtain papers referring to d-la, the following search terms were used: d-lact* AND (acidosis OR encephalopathy OR neuropathy). These databases were also searched for ME/CFS articles referring to acidosis (search terms: acidosis AND (“chronic fatigue syndrome” OR “myalgic encephalomyelitis”). Reference lists from articles obtained were manually screened to find other relevant references. Figure 1 shows the PRISMA flowchart for identification, screening and article exclusion.

#### Qualitative synthesis

Sixty-eight case reports were screened for inclusion in the qualitative synthesis (see Table 1). Case reports were screened in a two-step process. The first stage of this process involved identifying case reports that reported both d-lactate levels and neurological symptoms during an episode of d-la. Fifteen case reports were excluded at this stage due to an inability to obtain full-text or inadequate reporting of neurological symptoms and/or d-lactate levels. A serum d-lactate level of greater than 3.0 mmol/L has been proposed as a marker for d-la diagnosis [16]. However, using this criterion for exclusion was considered to be inappropriate when there were varying measurement methods used throughout the case reports. To reduce bias in case report selection, all cases that measured d-lactate and indicated that the patient’s d-lactate level was ‘high’ or above the ‘normal’ range, as stipulated by the authors and relevant measurement method, were included. Only one episode was excluded [17] when plasma d-lactate fell within the normal range according to the chosen method of measuring d-lactate defined within this case report. Across the episodes reviewed, there were considerable discrepancies between sampling and measurement methods (see Additional file 1: Table S1). A discussion paper on measurement issues and analyses is being compiled by our team and beyond the scope of the current review.

**Table 1:** d-Lactic acidosis case reports screened for qualitative synthesis

Episode code #	References	Included	Reasons for exclusion
1	[20]	N	d-Lactate measurement not specified
2	[17]	N	Plasma d-lactate within normal range
3	[21]	N	d-Lactate measurement not specified
4	[22]	Y	
5	[23]	N	d-Lactate measurement not specified
6	[24]	Y	
7	[25]	N	d-Lactate not d-la; No comparison between d-lactate and neurological symptoms
8	[26]	Y	
9	[27]	Y	
10	[28]	Y	
11	[29]	Y	
12a and 12b	[30]	Y	
13	[31]	Y	
14	[32]	Y	
15	[33]	Y	
16_1_ and 16_2_	[34]	Y	
17	[35]	Y	
18	[36]	Y	
19	[37]	Y	
20	[38]	Y	
21	[39]	Y	
22	[15]	N	d-Lactate levels not presented in relation to neurological symptoms
23	[40]	Y	
24	[41]	Y	
25	[42]	Y	
26*	[43]	Y	
27	[44]	Y	
28	[45]	Y	
29	[46]	Y	
30	[47]	Y	
31	[48]	Y	
32	[49]	N	Unable to obtain full-text
33	[50]	Y	
34_1_ and 34_2_	[51]	Y	
35	[52]	Y	
36*	[53]	Y	
37	[54]	Y	
38	[55]	N	Neurological symptoms not specified
39*	[56]	Y	
40	[57]	Y	
41	[58]	Y	
42*	[59]	Y	
43	[60]	Y	
44	[11]	Y	
45	[61, 62]	N	Same case for both references; d-lactate levels not specified
46	[63]	N	d-Lactate not measured
47a and 47b	[64]	Y	
48	[65]	Y	
49	[66]	Y	
50	[67]	Y	
51a and 51b	[68]	Y	
52	[69]	Y	
53	[70]	Y	
54	[71]	N	Unable to obtain English full-text
55	[72]	Y	
56	[73]	Y	
57	[74]	Y	
58a and 58b	[75]	Y	
59	[76]	Y	
60	[77]	Y	
61	[78]	N	d-Lactate measurement not specified
62	[79]	Y	
63	[80]	N	Neurological symptoms not specified
64	[16]	Y	
65	[81]	Y	
66	[82]	Y	
67	[83]	N	d-Lactate only measured during intervention

Subscript numbers (_1_ and _2_) indicate separate episodes for the same patient. The letters *a* and *b* identify different patient cases reported in the same reference. Episodes from non-SBS patients are marked with an asterisk. Episodes included in qualitative synthesis simultaneously reported at least one high D-lactate level (from blood or urine analysis) and documented neurological symptoms

During the second stage of screening, the remaining 53 case reports were independently assessed for quality by a team of three critical appraisers. Each article was assessed by two appraisers using the checklist developed by The Joanna Briggs Institute [18] based on the CARE Guidelines [19] established to improve the quality of reporting clinical cases. Appraisers rated (Yes, Unclear, No or N/A) on the eight items pertaining to (1) Demographic characteristics; (2) Patient history; (3) Current clinical condition; (4) Diagnostic tests; (5) Treatment/intervention; (6) Post-intervention clinical condition; (7) Adverse events; and (8) Take away lessons. Items 1–4 were prioritised as they were most relevant for the focus of this review. When comparing ratings across these four items, most articles (47/53, 90.1%) were rated as ‘Yes’ by both critical appraisers. For the remaining 6 articles, at least one reviewer provided a rating of ‘Unclear’ on an item. Discrepancies in ratings were discussed and the appraisers agreed that all articles adequately covered these priority domains and were deemed appropriate for inclusion in the qualitative synthesis.

Case reports that described multiple episodes (either for the same patient or different patients) were included as separate episodes if they met the eligibility criteria. From the 53 case reports, a total of 59 episodes were identified and included for qualitative synthesis. Each episode was reviewed with information about patient demographics, medical history, comorbid conditions, proposed triggers, neurological symptoms, non-neurological symptoms, d-lactate levels, l-lactate levels, anion gap, pH levels, microbial composition and treatment tabulated (Additional file 1: Table S1).

#### Determining ME/CFS and d-la symptom overlap

Reported d-la symptoms (neurological and non-neurological) were compared with ME/CFS International Consensus Criteria (ICC; [1]. Comorbid mood symptoms (including anxiety and depression) not required for ME/CFS diagnosis but frequently experienced by patients were also included for comparison with d-la presentations. d-la symptoms were classified as *‘matching’* ME/CFS symptoms or *‘ambiguous/other’*. d-la symptoms were only classified as *matching* if terminology was directly comparable to the symptoms described in the ICC (see Table 2). All other symptoms were categorised as *ambiguous/other.*

**Table 2:** Mapping overlap between myalgic encephalomyelitis/chronic fatigue syndrome (ME/CFS) and d-lactic acidosis (d-la) symptoms

ME/CFS International Consensus Criteria [1]	d-la symptoms mapped to ME/CFS criteria	
	Matching	Ambiguous/other
A. Postexertional neuroimmune exhaustion (compulsory)	A. Lethargy/fatigue	
B. Neurological impairments (at least one symptom from 3 of the 4 categories) B1. Neurocognitive impairments B2. Pain B3. Sleep disturbances B4a. Neurosensory and perceptual and B4b. Motor disturbances	B1. Encephalopathy/Mental confusion/disorientation/dazed/Concentration difficulties/Slow processing and responding to questions/slow speech B2. Headaches/Muscle pain B3. Drowsiness/sleepiness/somnolence B4a. Blurred vision B4b. Weakness/hypotonic (lowered muscle tone)/flaccidity/impaired gait (staggering/wide/ataxic/unsteady/instability)/ataxia (movement and co-ordination difficulties)/impaired balance	B1. Altered mental state/cortical dysfunction (e.g., disoriented to date, time, place and space)/delirium/blunted judgment/abnormal EEG B4a. Hallucinations (visual and auditory)/delusions/paranoid ideation B4b. Slowed cerebellar function/movement/dysiadochokinesia (difficulty performing rapid movement)/impaired reflexes/Neuropathy (fine motor coordination difficulties)/unable to grasp objects/Ptosis (eye drooping)/Asterixis (hand ‘flapping’/tremor)/Spasms: nystagmus (eye spasms)/opisthotonos (muscle spasms leading to hyperextended posture)/Bruxism
		*Speech symptoms:* Slurred and incoherent speech/dysarthria (speech pronunciation difficulties, weak muscles effecting speech)/thickened speech/ataxic speech (explosive—pauses between syllables)
		*Consciousness:* Altered/fluctuating/comatose/intermittent coma/stupor/induced sleep/depressed level of consciousness/obtunded/fluctuating from unrousable to alert
C. Immune, gastro-intestinal and genitourinary impairments (at least one symptom from 3 of the 5 categories) C1. Flu-like symptoms C2. Prone to viral infections C3. Gastro-intestinal abnormalities: nausea, abdominal pain, irritable bowel syndrome, bloating C4. Genitourinary symptoms C5. Sensitivities to food, medication, odours or chemicals	C3. Gastrointestinal symptoms*: Increased diarrhea/bowel movements Nausea/vomiting Diffuse abdominal pain	
D. Energy production/transportation impairments (at least 1 symptom) D1. Cardiovascular: orthostatic intolerance (inability to tolerate an upright position), postural orthostatic tachycardia syndrome, palpitations, arrhythmias, hypotension, dizziness, pallor D2. Respiratory: labored breathing, air hunger, fatigue of chest wall muscles D3. Thermostatic instability: lowered body temperature, cold extremities, marked diurnal fluctuations, sweating, episodic feverishness D4. Intolerance to temperature extremes	D1. Inability to stand/sit upright/Tachycardia (rapid heart rate)/Respiratory arrhythmia/Hypotension/low blood pressure/Dizziness/Pallor D2. Breathing difficulties: hyperpnoea (deep breathing)/dyspnoea (shortness of breath)/tachypnea (rapid breathing)/Kussmaul (deep and laboured)/breathlessness/hyperventilation D3. Body temperature changes (high or low)	D1. Bradycardia (slowed heart rate) D2. Respiratory acidosis and hypercarbic respiratory failure
Comorbid Mood and Behavioural Disturbances 1. Depressive symptoms 2. Anxiety symptoms	1. Unhappy/agitation/irritability 2. Anxiety	Irrational/unusual/disturbed behavior/aggressive/hostile/abusive/combative/uncooperative behavior/euphoria/aloofness
Uncategorized d-la symptoms		
		*Metabolic acidosis*
		*Other abnormalities:* dehydration/cravings (water, cigarettes)/excessive thirst Acute renal failure/hyperchloremic acidosis/liver dysfunction

ME/CFS broad category B. Neurological impairments are highlighted as the primary focus of this review and to show three subcategories of delineation under *ambiguous/other* symptoms (i.e., in accordance with specific ICC criteria (B1 – B4), speech/language symptoms, and level of consciousness)

* Gastro-intestinal symptoms associated with short bowel syndrome or the patient’s medical history were not included as symptoms of d-la. Only reports of a *change* in gastrointestinal symptoms were included

*ambiguous/other*: symptoms that were not clearly identified as consistent with ME/CFS presentation (see Table 2 for detailed symptom delineation), *d* *-la* d-lactic acidosis, *matching*: mapped overlap between ME/CFS and d-la symptoms, *ME/CFS* myalgic encephalomyelitis/chronic fatigue syndrome

#### Neurological symptoms

As neurological symptoms were the primary focus of this review, further categorisation was used to obtain more information about the types of neurological symptoms that accompany a d-la presentation. The *ambiguous/other* neurological symptoms were delineated into ME/CFS categories B1–B4, *speech* and *consciousness* subgroups (see Table 2). Reports of altered consciousness formed a distinct subcategory (*consciousness*) to identify the proportion of patients that presented with this more severe neurological symptom.

Speech and language impairment may have shared pathophysiology with other motor or neurocognitive disturbances. The ME/CFS ICC describes ‘slow speech’ without mentioning any other specific speech or language impairments [1]. Impaired information processing and word retrieval have been described as cognitive manifestations of ME/CFS, with speech therapy being a suggested treatment option [84]. The transient speech and language symptoms (e.g. dysarthria and/or slurred and incoherent speech) in d-la are likely to be overt behavioural manifestations of underlying muscle weakness and/or neurocognitive disturbances. However, without further information from each report, speech symptoms (excluding ‘slow speech’) were grouped as a subcategory for further investigation.

#### Conservative methodology

As highlighted by the aforementioned distinct classification of speech and language symptoms, we chose to pursue a conservative method of symptom categorisation. Several other *ambiguous* d-la symptoms were highly suggestive of ME/CFS and more likely to reflect discrepancies in terminology rather than different symptomatology per se. Inconsistent assessment and reporting of symptoms can reflect differences between patient demographics (i.e., age or sex), disciplines, and professional settings. This is particularly pertinent when considering comparisons between terminology used to describe chronic (i.e., ME/CFS) versus acute (i.e., d-la) presentations. For example, a patient presenting with ‘fluctuating consciousness’ or ‘comatose’ may preclude further neurological assessment and thus limit reporting of other covert symptoms. Similarly, the observable nature of motor and speech/language symptoms may be more frequently reported during an acute hospital presentation unlike some neurocognitive symptoms that require more specific testing and comparative measures to notice, for example a deterioration in memory, attention and clarity of thought. In another example, ‘slowed cerebellar function’ was used to describe d-la symptoms. This term is likely to reflect similar ME/CFS motor disturbances. However, the ICC does not specifically refer to ‘slowed’ movement, hence this symptom was classified as *ambiguous/other*. Consequently, our method of clinically comparing ME/CFS and d-la symptoms was conservative and chosen to ensure that symptom overlap was not inflated.

The presence of each reported d-la symptom was identified by episode number (see Table 1). This enabled frequencies and percentages to be calculated for both broad (A–D) and specific (B1–B4, C1–5, D1–4) ME/CFS ICC categories. Many episodes reported multiple neurological symptoms both within and between different subcategories i.e. *neurocognitive impairments* (B1) and *motor disturbances* (B4b). In these circumstances, each episode was only counted once for each specific subcategory. Likewise, an episode was only included once when calculating the presence of symptoms in each broad category, i.e. *neurological impairments* (B). Frequencies and percentages were calculated for each symptom category and delineated by available demographic details (age and sex). Episodes were classified as paediatric (≤17 years) or adult (≥18 years).

## Results

### Systematic summary of d-la episodes

A total of 59 episodes of d-la reported both neurological symptoms and d-lactate levels. The average patient age during d-la presentation was 29.9 years (*SD* = 21.0). Twenty-two paediatric (age range 10 months to 16 years, *M* = 7.1 years, *SD* = 4.5 years) and 37 adult (age range 18–60 years, *M* = 43.4 years, *SD* = 13.9 years) episodes were examined. There were 35 male and 23 female episodes with similar sex ratios documented for adult males (*n* = 20) and females (*n* = 17). Patient sex was not identified in one paediatric case. A predominance of male paediatric episodes (*n* = 15) were found compared with female paediatric episodes (*n* = 6). d-la episodes were primarily from patients with a history of SBS (55/59, 93.2%). The four patient episodes without SBS presented with propylene glycol intoxication [43], chronic pancreatitis [53], acute lymphoblastic leukaemia [56], and surgery error [59]. Table 3 summarises the frequency and percentage of reported d-la symptoms by age (paediatric and adult), sex (male and female) and total episodes.

**Table 3:** Frequency of episodes that reported *matching* and/or *ambiguous/other* d-lactic acidosis (d-la) symptoms as a function of age and sex

ME/CFS ICC	d-La symptom overlap with ME/CFS	Episode frequencies														
		Paediatric (≤17 years)							Adult (≥18 years)						Total	
		Male (15 episodes)		Female (6 episodes)		NI (1 episode)	Total (22 episodes)		Male (20 episodes)		Female (17 episodes)		Total (37 episodes)		(59 episodes)	
		*n*	%	*n*	%	*n*	*n*	%	*n*	%	*n*	%	*n*	%	*n*	%
A. Postexertional neuroimmune exhaustion	Matching	8	53.3	1	16.7	–	9	40.9	2	20.0	4	23.5	6	16.2	15	25.4
	Ambiguous/other	–	–	–	–	–	–	–	–	–	–	–	–	–	–	–
B. Neurological impairments	Matching	12	80.0	6	100	1	19	86.4	18	90.0	15	88.3	33	89.2	52	88.1
	Ambiguous/other	11	73.3	2	33.3	1	14	63.6	17	85.0	14	82.4	31	83.8	45	76.3
	*Ambiguous/other B1*–*B4*	3	20.0	1	16.7	1	5	22.7	11	55.0	6	35.3	17	45.9	22	37.3
	*Speech/language*	7	46.7	2	33.3	1	10	45.5	11	55.0	9	52.9	20	54.1	30	50.8
	*Consciousness*	5	33.3	–	–	–	5	22.7	5	25.0	3	17.6	8	21.6	13	22.0
C. Immune, gastrointestinal, genitourinary impairments	Matching	3	20.0	–	–	–	3	13.6	6	30.0	4	23.5	9	24.3	12	20.3
	Ambiguous/other	–	–	–	–	–	–	–	–	–	–	–	–	–	–	–
D. Energy production/transportation impairments	Matching	8	53.3	3	50.0	–	11	50.0	7	35.0	2	11.8	9	24.3	20	33.9
	Ambiguous/other	–	–	–	–	–	–	–	2	10.0	–	–	2	5.4	2	3.4
Mood/behavior	Matching	2	13.3	1	16.7	–	3	13.6	2	10.0	3	17.6	5	13.5	8	13.6
	Ambiguous/other	6	40.0	–	–	–	6	27.3	4	20.0	3	17.6	7	18.9	13	22.0
Uncategorized d-la symptoms																
	*Metabolic acidosis*	15	100	6	100	1	22	100	20	100	16	94.1	36	97.3	58	98.3
	*Other abnormalities*	4	26.7	–	–	–	4	18.2	6	30.0	2	11.8	7	18.9	11	18.6

Percentages were calculated from fractions of the number of episodes that reported relevant symptoms (*n*) against the number of possible episodes (noted in column subheadings) within each sex and age category. ME/CFS broad category B Neurological impairments are highlighted as the primary focus of this review. The *ambiguous/other* symptoms are further delineated into three subcategories (ICC criteria B1–B4, speech/language symptoms, and level of consciousness; see Table 2 for explanations). In each subcategory the same episode code number can be shown several times to represent multiple symptoms during each d-la episode. See Additional file 2: Table S2 for an expansion of these results showing episode code numbers that were included for each symptom category

*Ambiguous/other* symptoms that were not clearly identified as consistent with ME/CFS presentation (see Table 2 for detailed symptom delineation), *B1–B4* neurocognitive impairments, pain, sleep disturbances, neurosensory and perceptual, motor disturbances, *d*-*la* d-lactic acidosis, *ICC* International Consensus Criteria, *Matching* mapped overlap between ME/CFS and d-la symptoms, *ME/CFS* myalgic encephalomyelitis/chronic fatigue syndrome, *NI* sex not identified

Table 3 shows some evidence of shared symptomatology across each broad ME/CFS ICC category. The highest percentage of overlap was found for neurological symptoms. Other symptoms specific to d-la were also frequently reported (e.g., metabolic acidosis). ME/CFS symptom categories are discussed sequentially to examine similarities with d-la symptoms.

### Overlap between d-la and ME/CFS symptoms

#### Post-exertional neuroimmune exhaustion

This ME/CFS symptom describes a chronic pattern of excessive and disproportionate fatigue upon exertion. This is the core compulsory symptom of ME/CFS [1]. In the context of the chronicity of ME/CFS symptoms, it is difficult to directly compare this pattern of post-exertional exhaustion with an acute presentation of d-la. Nevertheless, one quarter of patients reported symptoms of lethargy and fatigue during a d-la episode (15/59, 25.4%). In contrast, all ME/CFS patients experience fatigue and lethargy as it is a required diagnostic criterion. The lower frequency of fatigue reported in d-la, may accurately reflect characteristic distinctions between the two conditions. Alternatively, an acute presentation of d-la can include fluctuating levels of consciousness and hence symptoms of fatigue may be less relevant and/or underreported within this emergency hospital setting.

#### Neurological impairments

Episodes reviewed in this qualitative synthesis required neurological symptoms to be reported (as an inclusion criterion), accordingly, all episodes of d-la reported at least one neurological impairment. The majority of neurological symptoms that were reported overlapped with ME/CFS symptomatology (52/59, 88.1%). The frequencies of *matching* ME/CFS neurological symptoms were similar when comparing paediatric (19/22, 86.4%) and adult (33/37, 89.2%) episodes as well as male (30/35, 85.7%) and female (21/23, 91.3%) episodes. *Ambiguous/other* neurological impairments (e.g., altered mental state or cortical dysfunction) were also frequently reported (45/59, 76.3%). The more severe neurological symptom of an altered level of consciousness was reported in 13 episodes (22.0%). Five case reports documented the patient’s altered consciousness as the only neurological symptom during the d-la episode. The remaining reports described additional neurological symptoms and a deterioration in symptoms affecting consciousness.

**Fig. 2:**
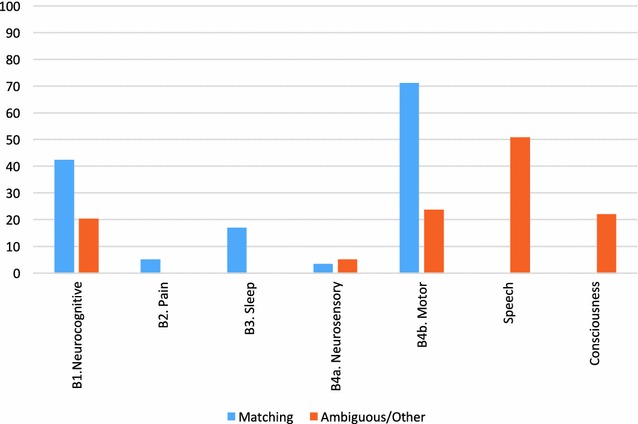
Percentages of d-lactic acidosis (d-la) episodes that reported ME/CFS *matching* and *ambiguous/other* neurological impairments. Total percentages are reported for neurocognitive symptoms (B1), pain (B2), sleep disturbance (B3), neurosensory and perceptual disturbances (B4a), motor disturbances (B4b), speech symptoms, and altered consciousness. N.B. The same episode may be represented multiple times for both *matching* and *ambiguous/other* symptom groups across all neurological impairment subcategories

When considering more specific types of neurological impairments, motor disturbance (B4b) was the most frequently reported *matching* ME/CFS neurological symptom (42/59, 71.2%, see Fig. 2). This was notably higher than the other neurological symptoms (B1. Neurocognitive = 25/59, 42.4%, B2. Pain = 3/59, 5.1%, B3. Sleep = 10/59, 16.9%, B4a. Neurosensory and Perceptual = 2/59, 3.4%). Common motor disturbances in ME/CFS include muscle weakness, clumsiness, balance and coordination difficulties [84]. The ICC noted that the presence of balance and gait instabilities are more frequently observed in severe cases [1]. *Ambiguous/other* neurocognitive, neurosensory, perceptual and motor disturbances were reported in 37.3% of total d-la episodes (22/59). Within these 22 episodes, 90.9% (20/22) of episodes simultaneously reported at least one *matching* neurological symptom akin with ME/CFS diagnostic criteria. Therefore, there was considerable overlap between *matching* symptoms and *ambiguous/other* neurological symptoms.

Approximately half of d-la episodes reported impairments in speech (30/59, 50.9%). Notably, all episodes that reported speech and language impairments also reported at least one other ME/CFS-*matching* neurological impairment, which may reflect the shared pathophysiology that underlies the behavioural manifestation of overt speech symptoms.

#### Immune, gastrointestinal and genitourinary impairments

The majority of d-la episodes were from patients with SBS (55/59, 93.9%). As such, these patients had a history of extensive gastrointestinal abnormalities. The case report of the patient with leukaemia [56] was the only episode of d-la that did not report any gastrointestinal symptoms during the acute stage or prior history. This episode was also an exception as it was the only episode of d-la without metabolic acidosis (discussed below). A *change* in ME/CFS-*matching* gastrointestinal symptoms associated with the d-la presentation was only reported in 22.0% of the total episodes (13/59). These changes included an increase in diarrhoea, nausea, vomiting and/or abdominal pain/distension.

Immune or genitourinary impairments (*matching* or *ambiguous/other*) were not specifically reported during d-la episodes. Conversely, immune symptoms are a primary component of ME/CFS as a neuro-immune condition with evidence of immune abnormalities [85] and autoimmune mechanisms [86].

#### Energy production and transportation impairments

ME/CFS-*matching* energy production and transportation impairments were reported in 33.9% (20/59) of total d-la episodes. These symptoms were more frequently reported in male (15/35, 42.9%) compared with female episodes (5/23, 21.7%; see Table 3). *Ambiguous/other* cardiovascular (bradycardia) and respiratory symptoms (respiratory acidosis and hypercarbic respiratory failure) were documented during two adult male episodes (2/59, 3.4%).

#### Comorbid mood and behavioural disturbances

Mood disturbances are not included in ME/CFS diagnostic criteria. However, patients with ME/CFS frequently experience comorbid anxiety and depressive symptoms [1, 87]. *Matching* mood (depressive and anxiety) symptoms were reported in 13.6% of d-la episodes (8/59). *Ambiguous/other* ME/CFS mood and behavioural disturbances were described in 22.0% of d-la episodes (13/59). The higher frequency of *ambiguous/other* mood and behavioural disturbances seen in paediatric male (6/15, 40.0%) compared to paediatric female episodes (0/6) may reflect the tendency for boys to externalise behaviours more than girls [88].

#### Other symptoms (non-ME/CFS)

Metabolic acidosis as defined by blood pH values below 7.35 [89] and/or as stipulated in each case report based on patients’ anion gap, was confirmed in all except one episode of d-la (58/59, 98.3%). Metabolic acidosis occurs when there is a decrease in serum bicarbonate, excess hydrogen ions and, commonly, a lower pH value suggestive of acidosis [90]. However, in some situations an underlying metabolic acidosis can be reflected in higher pH values that are indicative of alkalosis but are secondary to a metabolic acidosis, sometimes referred to as a compensatory process [90]. In Mendu et al. [56] the authors described the normal serum pH values (7.35–7.45) as a “compensated metabolic acidosis” due to simultaneous higher l-lactate levels observed in this patient (p. 90). Metabolic acidosis is a primary marker of d-la but is not described in ME/CFS diagnostic criteria. Blood pH values are not routinely measured in ME/CFS, therefore, the symptomatic overlap cannot be determined.

Other abnormalities such as dehydration, cravings and excessive thirst were infrequently reported in the d-la episodes (9/59, 15.3%). Acute renal failure, hyperchloremic acidosis and liver dysfunction were reported in three separate episodes (3/59, 5.1%).

## Discussion

**Fig. 3:**
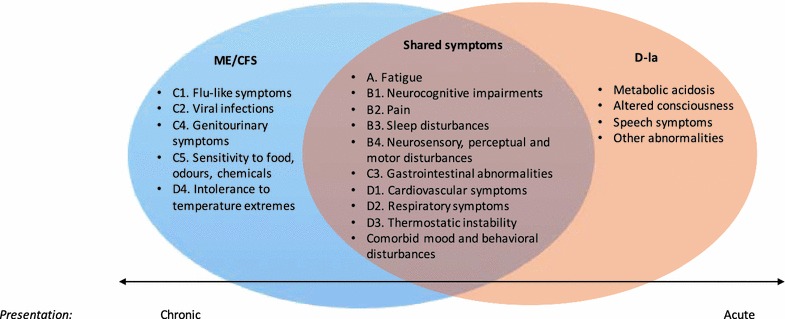
Proposed continuum of d-lactic acidosis and ME/CFS symptoms. A continuum showing, from *left* to *right*; *distinct* myalgic encephalomyelitis/chronic fatigue syndrome (ME/CFS) symptoms, *shared* and *distinct* d-lactic acidosis (d-la) symptoms. Continuum also shows *chronic* compared to *acute* presentations

Examples of *matching* ME/CFS and d-la symptoms were found throughout the d-la case reports. More than 96.6 per cent (57/59) of d-la episodes reported at least one *matching* ME/CFS symptom. Whilst there was considerable overlap, some symptoms of both ME/CFS and d-la were distinct. Figure 3 provides an overview of shared and distinct symptoms in these acute and chronic clinical conditions.

This qualitative synthesis has confirmed that the type of neurological impairments reported during d-la episodes are similar to those experienced by ME/CFS patients. However, the most commonly reported motor disturbances in d-la are considered a more severe presentation within ME/CFS [1]. This may reflect differing pathophysiology or alternatively may support a proposal for both conditions to lie on a continuum. ME/CFS may fall at one end as a chronic condition with fluctuating severity and d-la at the other extreme as an exacerbation of an acute presentation (see Fig. 3). The fluctuating neurological symptoms that present in both d-la and ME/CFS may vary in severity and the corresponding treatment response [66]. Htyte et al. [40] described these transient symptoms as “usually mild and self-limiting in patients with normal renal function” (p. 1435), highlighting the individual variation in presentation and reporting of symptoms with less severe symptoms unlikely to prompt acute emergency care.

Some key areas of disparity between d-la and ME/CFS symptoms related to immune impairments and metabolic acidosis. These results may accurately reflect pathophysiological differences between the two conditions. Alternatively, some other plausible explanations warrant consideration. The lack of reports relating to specific immune symptoms in d-la may be related to symptom prioritisation during an acute presentation. Reports of bacterial infection preceding d-la onset, bacterial overgrowth during the d-la episode and response to antibiotic treatment (see Additional file 1: Table S1), suggest that immune dysfunction may still be relevant for d-la patients.

Without measurement of blood pH levels the prevalence of metabolic acidosis in ME/CFS is unknown. Other research raises questions about the possibility of similar mechanisms of metabolic acidosis (or the compensatory acidosis described above) in ME/CFS. Alkalosis in skeletal muscles may result in a compensated acidosis in the blood, precipitating hyperventilation [91]. This theory has been proposed from evidence of hyperventilation in patients with ME/CFS [92] and an inverse association between skeletal muscle pH and cerebral blood flow [91]. Compared to sedentary controls, ME/CFS patients have higher skeletal muscle pH at rest [93] and at recovery after exercise [93]. Alkalosis in skeletal muscle has been proposed as a mechanism effecting orthostatic and neurocognitive ME/CFS symptoms [91]. Blood acidosis can also directly alter the function of cellular membranes [91], therefore, our current understanding of the mechanisms involved remain rudimentary. Routine assessment of blood pH levels in ME/CFS will ascertain the prevalence of metabolic acidosis/alkalosis for this clinical population.

### Limitations

These results need to be considered with an awareness of potential methodological limitations. Firstly, the inclusion criteria for selected case reports in this review may have been problematic. Although unavoidable, the requirement of neurological symptoms during d-la episodes may have increased reporting bias during this review process leading to an exaggerated focus on neurological symptoms. However, the effect of this limitation may be moderated when considering the high percentage of case reports meeting both the eligibility criteria of describing neurological symptoms and d-lactate measurement during the episode (80.0%).

Findings from this qualitative review are also limited by the lack of standardised procedures used when reporting symptoms in case reports. Differences in assessment procedures and terminology used for reporting neurological symptoms may impede accurate interpretation. Some *ambiguous/other* symptoms described as distinct may share similar pathophysiology. This may be particularly pertinent for speech symptoms. On the one hand, the results may underestimate the level of overlap based on the conservative classification of symptoms. Alternatively, the breadth of ME/CFS symptoms included in the ICC diagnostic criteria may inflate the findings. Reliance on qualitative symptom report comparisons only provide a preliminary guide to shared symptomatology. Whilst useful for theoretical purposes it is insufficient to draw confirmatory conclusions.

### Implications

Mindful of these limitations, the proposal of a continuum of acute and chronic encephalopathy related to d-lactate warrants further investigation. Within d-la, several authors have proposed that the level of acidosis and associated encephalopathy may result in differing severity and either an acute or chronic presentation [27, 28, 32]. A subclinical elevation of d-lactate has been reported in SBS patients and diverse populations [94]. Higher d-lactate levels were recorded in 2.8% of randomly selected hospital patients [40]. Minimal details were provided about this sample other than noting that 40% of these patients did not have a history of gastrointestinal surgery [40]. Higher levels of d-lactate have also been recorded in response to trauma or infection [95]; and in patients with diabetes compared with healthy controls [96]. Thornalley et al. [97] showed positive correlations between the level of d-lactate and duration of diabetes. They found that the duration of disease was positively associated with retinopathy, neuropathy and nephropathy complications of diabetes. The relevance of d-lactate for diverse presentations is currently unknown.

Even within SBS populations, d-la has been under-recognised and frequently misdiagnosed [9]. Misdiagnosis is complicated by issues related to accurate and efficient measurement of d-lactate. A further diagnostic complication related to the clinical presentation of d-la is that the neurological manifestations can present without gastrointestinal complications or a change in gastrointestinal symptoms. Less than one-quarter of d-la episodes analysed in this review described a worsening of gastrointestinal symptoms. Therefore, it is plausible that clinicians may focus on the neurological presentation and overlook the underlying gastrointestinal mechanism. The case report from Scully et al. [70] highlighted this when the 16-year old male patient was first treated by a psychiatrist with lithium carbonate for suspected bipolar disorder and tested for illicit drug use before being diagnosed with d-la. The patient presented with aggression, somnolence and weight loss without current gastrointestinal symptoms, although had an abdominal trauma one year prior that required short-bowel surgery [70]. The presence of neurological symptoms in the absence of current gastrointestinal symptoms may lead to frequent misdiagnoses. The proposed mechanisms of d-la (i.e., carbohydrate malabsorption and related bacterial overgrowth [32, 36]) may have relevance for patients presenting with neurological symptoms but without an observable change in gastrointestinal symptoms.

Carbohydrate malabsorption is not exclusive to SBS populations and can vary in severity. Altschule et al. [98] found that d-lactate was more slowly metabolised in patients with schizophrenia, manic-depression and psychosis compared with healthy controls. Even earlier studies have shown increased lactate after fructose or glucose ingestion and disturbed lactate metabolism after exercise within these populations [98], suggesting difficulties with carbohydrate metabolism. Within ME/CFS, carbohydrate restriction (e.g., avoidance of sugars and grains) may be advantageous [99, 100]. Whilst there is minimal empirical support, clinical reports suggest that dietary triggers can exacerbate symptoms and that some patients benefit from dietary exclusions [99]. The response to treatment for small intestine bacterial overgrowth (SIBO) in ME/CFS patients [101], suggests that carbohydrate malabsorption may be relevant for a subgroup of this population. An analysis of mechanisms involved in d-la is provided to help identify shared pathophysiology between d-la and ME/CFS.

## Part B. Narrative review

### Proposed mechanisms in d-lactic acidosis

**Fig. 4:**
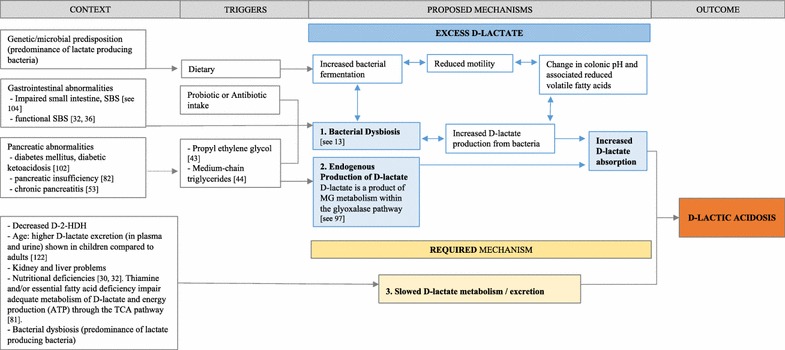
Overview of mechanisms in d-lactic acidosis. *Legend.* Summary of contextual factors, triggers and proposed mechanisms leading to d-lactic acidosis (d-la). A presentation of d-la requires an increase in d-lactate absorption. The proposed mechanisms of increased d-lactate production can be through (1) bacterial dysbiosis and/or (2) endogenous production of d-lactate. Simultaneous to the increased absorption, patients who present with d-la also require (3) slowed metabolism of d-lactate. Hence, the d-lactate production exceeds the body’s metabolic and/or excretion capacity. Short bowel syndrome (SBS); d-2-hydroxy acid dehydrogenase (d-2-HDH); adenosine triphosphate (ATP); tricarboxylic acid (TCA)

Understanding d-la involves firstly establishing the reason for increased d-lactate levels before examining proposed neurological mechanisms. Figure 4 summarises the contextual factors, triggers and proposed mechanisms leading to d-la. The presentation of d-la requires both an increase in d-lactate absorption that exceeds the metabolic and/or excretion capacity of the patient.

#### Bacterial dysbiosis

Bacterial dysbiosis (i.e., an imbalance in commensal bacteria [2]) has been suggested as the primary mechanism influencing d-la presentation in SBS populations. The dysbiosis is distinguished by an increased colonisation of lactic acid-producing bacteria, particularly bacteria that produce d-lactate (e.g., *Lactobacillus fermenti, L acidophilus,* and *Streptococcus*; see review by Petersen [13]). An overgrowth of *Lactobacilli* has been identified in SBS patients with increased d-lactate levels [26, 28, 37, 47, 64, 68, 72, 74, 75, 103]. This dysbiosis has been proposed as a result of an impaired small intestine, either due to congenital causes, surgery for midgut volvulus, gangrene or inflammatory bowel disease [104]; functional SBS and carbohydrate malabsorption [32, 36]; or colonic stagnation [62]. With reduced absorptive capacity of the small intestine, malabsorbed carbohydrates are more likely to enter the colon and provide fuel for colonic bacteria leading to increased bacterial fermentation [54, 81]. Increased bacterial fermentation can further reduce bowel motility [31], alter colonic pH and change the level of bacterial metabolites. This can include a reduction in volatile fatty acids [26] and increased d-lactate production [shown in 26, 54, 64, 66, 75, 105].

Dietary, probiotic and antibiotic intake have preceded bacterial dysbiosis and d-la presentations. Some episodes of d-la have been triggered by increased sugars/carbohydrate (e.g., [31, 42, 66]) or a change from parenteral to oral intake (e.g., [48, 70]). In patients with bacterial dysbiosis, diet and probiotic supplementation can increase bacterial fermentation and further alter bacterial composition. It appears that the type of diet or probiotics can influence d-lactate production in either a beneficial or detrimental manner. Whilst antibiotics are commonly used as a treatment for d-la, indiscriminate and inappropriate use of antibiotics has also been shown to precede and potentially trigger d-la [36]. The way antibiotics alter bacterial composition may lead to further dysbiosis and an increased d-lactate production in vulnerable patients.

Although bacterial dysbiosis is the primary mechanism used to explain the occurrence of d-la, enteric microbial composition was only measured prior to treatment for 21 of the 59 episodes screened for the qualitative review (35.6%). More consistent measurement of the gut microbiome may add clarity to d-la etiology and individual treatment.

#### Slowed d-lactate metabolism/excretion

Whilst it is beyond the scope of this review to explain lactate metabolism (see [13, 106]) a brief overview of d-lactate metabolism in relation to d-la is provided. Humans can effectively metabolise large amounts of d-lactate. Hove and Mortensen [15] confirmed that humans have the enzyme d-2-hydroxy acid dehydrogenase (d-2-HDH) to enable conversion of d-lactate to pyruvate. Certain conditions such as increased oxalate and low pH can inhibit the activity of d-2-HDH enzymes, as shown in animal tissue [107]. The kidney and liver have the highest concentrations of d-2-HDH. Therefore, kidney and liver impairments can reduce effective metabolism of d-lactate indicated by d-lactate accumulation in patients with renal dysfunction [40] and liver cirrhosis [108]. The presence of adequate d-2-HDH is required for d-lactate metabolism.

Colonic bacteria can be involved in both lactate production and excretion during pyruvate metabolism. Human and some bacterial mitochondria have the enzyme dl-lactate racemase which enables conversion between d-and l-lactate [15]. For example, *Lactobacillus* species are common producers of lactate but the ratio of d- and l-lactate production and the direction of conversion is dependent on the species (see [109]). Therefore, impaired colonic metabolism of d-lactate may also be a consequence of bacterial dysbiosis. Colonic flora that is predominated by lactate-producing bacteria and a reduced capacity to convert lactate to short chain fatty acids (SCFA) will result in less SCFA and reduced metabolism of d-lactate [13].

Impaired metabolism of consequential d-lactate accumulation is required for the presentation of d-la [25]. It may be beneficial to categorise patients into lactate accumulators vs non-lactate accumulators. When examining bacterial composition in a sample of SBS patients, Mayeur et al. [110] showed that some patients preferentially accumulated d-lactate compared with l-lactate, suggesting the influence of bacterial composition on d-lactate profiles. The d-lactate accumulators were more likely to experience encephalopathy symptoms. Therefore, multiple factors including increasing bacterial d-lactate production, other endogenous production of d-lactate, nutritional status and altered d-lactate metabolism will effect d-lactate accumulation and the clinical presentation of an episode of d-la.

### Proposed neurological mechanisms in d-lactic acidosis

**Fig. 5:**
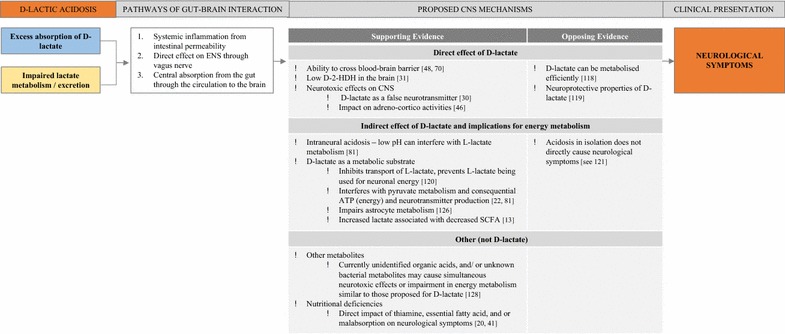
Proposed mechanisms of gut–brain interaction leading to neurological symptoms observed in d-lactic acidosis. Supporting and opposing evidence for proposed central nervous system (CNS) mechanisms are categorised according to direct effects of d-lactate, indirect effects of d-lactate and other possible mechanisms unrelated to d-lactate. Abbreviations: enteric nervous system (ENS); d-2-hydroxy acid dehydrogenase (d-2-HDH); adenosine triphosphate (ATP); short chain fatty acids (SCFA)

Metabolic acidosis and increased d-lactate levels are synonymous with the presentation of d-la. However, neither condition can predict the development of neurological symptoms. Acidosis can occur without associated neurological symptoms and in reverse, encephalopathy can be present without the accompanying acidosis [13]. Similarly, whilst increased d-lactate levels are required in d-la, the presence of high d-lactate is not the sole cause or determinant of neurological symptoms. Some studies have shown a temporal association between d-lactate level and symptom onset [30] as well as severity [103]. However, this has not always been replicated (e.g., [48]). Other factors must also be required because higher d-lactate levels have been shown in patients with SBS and other gastrointestinal disorders but without concurrent encephalopathy [111]. These inconsistencies suggest that there are several possible direct and indirect mechanisms responsible for the neurological manifestations in d-la (see Fig. 5).

#### Possible pathways of gut–brain interaction

Three pathways have been proposed to explain how excess d-lactate production in the colon can impact neurological symptoms [112]. Firstly, a colonic environment with lowered pH and high lactate levels can increase intestinal permeability (i.e., aberrations in the mucosal lining of the gastrointestinal tract [113, 114]) and result in systemic inflammation. For example, in ruminants, preliminary findings showed that lactic acidosis (lowered pH and increased faecal lactate) was significantly associated with increased tumor necrosis factor-alpha (TNF-α [115]). Translocation of luminal content, including endotoxins, to bodily fluid or tissue may result in an increased immune response and associated neurological sequelae [116].

A second pathway of gut–brain interaction is through neural mechanisms. The bidirectional communication between the enteric nervous system (ENS) and central nervous system (CNS) via the vagus nerve can regulate or dysregulate neurotransmitter production [117]. Bacterial dysbiosis can have both direct and indirect effects on neurotransmitter production and associated neurological symptoms (see [2]). Dahlquist et al. [30] suggest that the effect of d-lactate on neurotransmitter production is one possible explanation for the temporal association between neurological symptoms and d-lactate levels observed during some d-la episodes. Alternatively, d-lactate may act by reducing neuronal energy metabolism as explored below.

Thirdly, excess d-lactate can act centrally in the colon and be absorbed and transported from the gut to the brain through the circulation. Hanstock et al. [112] provided support for this being a primary mode of action finding significant associations between plasma and colon/caecum d-lactate levels in rats. d-Lactate can cross the blood–brain barrier with evidence of d-lactate in both circulating plasma and cerebrospinal fluid in human case reports of d-la [48, 70]. Whilst murine models show reduced uptake of d-lactate compared with l-lactate within the brain [112], slowed metabolism due to low d-2-HDH in the brain may explain the subsequent neurological symptoms [31]. An increased d-lactate level within the brain may exert direct and indirect effects on the CNS.

#### Proposed central nervous system mechanisms

##### Direct effect of d-lactate

As a substrate on its own, the direct neurotoxic effect of d-lactate is questionable and unlikely. Intravenous infusion of d-lactate in healthy males did not result in evidence of neurological symptoms [118]. However, at substantially higher levels, as shown in propylene glycol intoxication [43], or in combination with nutritional deficiencies [30], direct neurotoxic effect may be possible. d-Lactate may act as a “false neurotransmitter” [30, p. 145]. Similar fluctuations in biochemistry concurrent with non-specific EEG abnormalities during an adult episode of d-la may support this proposal [74]. However, this remains propositional without further evidence of the precise mechanisms involved.

The neuroprotective properties of d-lactate have also been described raising doubt about the neurotoxicity of d-lactate alone. Castillo et al. [119] showed that both l- and d-lactate can exert neuroprotective properties in a male mouse model of cerebral ischemia (stroke). Unexpectedly, they found that d-lactate showed near equivalent neuroprotective properties (i.e., reduced cell death, less damage observed on behavioural measures) to that shown with l-lactate. Unlike earlier findings, their results indicated that d-lactate can be metabolised by the rodent brain. This raises the possibility that d-lactate may also be able to be metabolised within human cerebral tissue. Notably this evidence is from a stroke animal model and requires investigation before generalising to d-la. It further highlights that d-lactate alone may not be neurotoxic but can play deleterious roles in certain environments when combined with other necessary conditions (e.g., nutritional deficiencies, excess glutamate, or mitochondrial toxicity) to produce the encephalopathy observed in d-la.

It has also been proposed that d-lactate can inhibit l-lactate transportation [120]. Considering that l-lactate can have an inhibitory effect on the adrenal cortex [46], it could be surmised that excess d-lactate may reduce available l-lactate and consequentially increase adreno-cortico activity. Further investigation of this mechanism is warranted. Growing evidence provides support for the role of d-lactate in energy metabolism.

##### Indirect effects of d-lactate and implications for energy metabolism

Previous research has proposed that d-lactate levels can reduce the pH balance within the brain and impede neurological processes [81]. Low pH can interfere with l-lactate metabolism [42]. However, in animal models it appears that the intraneural acidosis itself is not the primary mechanism at play (see [121]). Similarly in clinical d-la cases, in isolation the acidosis does not directly cause neurological symptoms [32]. Reduced d-lactate is more closely related to clinical improvement than neutralizing pH [39]. Bongaerts et al. [122] also showed that there was not a direct correlation between d-la and acidosis. Rather than intraneural acidosis, the competing role of l- and d-lactate for cerebral metabolism is a preferred explanation [121].

Pairing lactate and glutamate in the first in vivo studies in male rats demonstrated the neuroprotective properties of l-lactate and neurotoxic properties of d-lactate [120]. The mechanism appears to be related to d-lactate’s influence on energy metabolism. When d-lactate was combined with glutamate, larger cortical lesions were produced [120]. This result suggests that d-lactate inhibits transport of l-lactate and prevents l-lactate being used for neuronal energy. Ros et al. [120] findings indicate the compounding neurotoxic effects of d-lactate when combined with excess glutamate. In a comparable murine study, Cassady et al. [121] showed that compared to d-lactate, l-lactate is the preferred substrate for cerebral energy. d-Lactate increased the excretion of amino acids and therefore was unable to act as an efficient metabolic substrate [121]. Variable levels of glutamate and other amino acids may explain why some people experience neurological symptoms and others do not.

Overlap between symptoms of pyruvate metabolism disorders and d-la presentation suggest that d-lactate can interfere with pyruvate metabolism and consequently reduce energy (adenosine triphosphate: ATP) and neurotransmitter production [81, 123]. Lower levels of a primary enzyme required for pyruvate metabolism, pyruvate dehydrogenase, have been found in the healthy cerebellum [124]. An increased d-lactate level that further impedes pyruvate metabolism may explain the predominance of motor/cerebellar symptoms observed in d-la [81]. Pyruvate metabolism abnormalities can interfere with optimal mitochondrial energy production [123]. This has potentially more revealing implications for organs that require greater energy, such as the brain and heart [123]. Ling et al. [123] found that d-lactate was an inadequate metabolic substrate and produced lower respiration in murine brain and heart mitochondria, however equivalent respiration rates were shown in liver tissue. d-Lactate was shown to inhibit l-lactate and pyruvate metabolism in brain and heart tissue.

The inhibition of l-lactate by d-lactate effects memory formation in day old chickens [125, 126]. The impaired metabolism may not only occur in neuronal cells as suggested by Baker and Edwards [125]. Gibbs and Hertz [125] results revealed that the inhibitory action of d-lactate occurs in astrocytes either through an extracellular effect or an intracellular effect impairing pyruvate metabolism in astrocytic mitochondria. Astrocytes play a primary role in maintaining homeostasis in the brain, including regulating glutamate use and removal, neuronal energy, and neuronal pH [127]. Gibbs and Hertz’s [125] results demonstrated that the presence of d-lactate prior to a learning task prevented memory formation, but memory loss was delayed by 20 min when d-lactate was injected 10 min after the learning task. The authors suggest that impaired memory formation in day-old chicks is comparable with the encephalopathy observed in d-la. Therefore, similar mechanisms may be responsible for neurological symptoms in the mammalian brain.

##### Other possible mechanisms, not d-lactate

Most research has focused on d-lactate’s role in precipitating the neurological symptoms observed in d-la. However, other metabolites and nutritional deficiencies may play causative and/or contributory roles in the encephalopathy observed in this condition. The suggestion to investigate other causative factors has been supported by evidence of increased d-lactate levels in healthy populations [14, 118] and poor direct association between d-lactate level and clinical symptoms [13]. Colonic bacteria can produce several other metabolites (including alcohol, organic acids, amines, mercaptans, and aldehydes) that may exert neurotoxic or neuromodulating effects by influencing neurotransmitter production [128]. Indirectly, higher d-lactate produced by an increased abundance of lactic-acid producing bacteria may reduce the presence of other bacteria that metabolise SCFAs. The reduced availability of SCFA can impact energy metabolism and neurotransmitter production [13]. Currently unidentified organic acids or unknown bacterial metabolites may cause simultaneous neurotoxic effects or impairment in energy metabolism similar to those proposed for d-lactate [20].

As alluded to earlier, the nutritional deficiencies commonly present in SBS populations may exacerbate the clinical presentation [20]. Adequate nutrition is required for brain development with nutrient deficiency or insufficiency having both broad and specific effects on regions of the brain and neural functioning (see Georgieff [129]). Within d-la, nutritional deficiencies may directly impact neurological symptoms or the reduced availability of nutritional substrates may alter d-lactate metabolism, clearance or utilization within the brain. Hudson et al. [40] presented a case report of a patient with d-la and thiamine deficiency where thiamine supplementation effectively resolved neurological symptoms. Interestingly in Wernicke encephalopathy, another condition that presents with acute confusion, delirium and ataxia, thiamine deficiency is responsible for these neurocognitive symptoms that resolve once adequate thiamine levels are restored (see Latt and Dore [130]). Thiamine is required for effective pyruvate metabolism in the brain, particularly within the cerebellum, hence thiamine deficiency may contribute to the encephalopathy seen in some patients with d-la.

### Summary

There is more support for the indirect effect of d-lactate interfering with energy metabolism in the CNS compared with the direct neurotoxic effects of d-lactate. Multiple mechanisms may be at play. Evidence of the inhibitory action of d-lactate on utilisation of l-lactate in neural cells and astroglia appears a particularly pertinent mechanism that may explain the neurological symptoms observed in d-la. The relevance of other bacterial metabolites remains in question. The vulnerability of certain individuals related to predisposing genetic, microbial factors or nutritional status that influence d-lactate production and/or adequate excretion/metabolism is likely to contribute to the presentation of d-la.

### What is the relevance for ME/CFS?

Whilst d-lactate levels have not been specifically measured in ME/CFS patients, elevated lactate levels within ventricular cerebrospinal fluid have been observed. Significantly higher levels of ventricular lactate were recorded in the ME/CFS patient group compared to both generalized anxiety disorder (GAD) and control groups. From this small sample of 16 CFS patients, 10 patients had high ventricular lactate levels, indicated by lactate levels above 2 standard deviations above control mean whereas the remaining 6 participants had equivalent lactate levels to both the GAD and healthy control groups. This distinction between clinical and control groups gives promise for ventricular lactate being a potential biomarker useful for establishing ME/CFS subgroups. Interestingly lactate level was not associated with any other demographic or clinical variables, including severity of illness. Notably, clinical measures of anxiety, depression, fatigue, sleep quality and fibromyalgia were used as outcome variables but cognitive symptoms were not measured. More detailed analysis of associations between objective neurocognitive symptoms and ventricular lactate level would be valuable. The authors explained the potential mechanisms related to mitochondrial dysfunction and/or oxidative stress that precede reduced cerebral blood flow which in turn upregulates anaerobic glycolysis and consequential lactate accumulation [131]. Mitochondrial dysfunction or increased oxidative stress may have bacterial and/or viral origins, or be related to underlying gastrointestinal abnormalities.

### Gastrointestinal abnormalities

Examination of gastrointestinal abnormalities in ME/CFS indicate some similarities between d-la mechanisms and ME/CFS pathophysiology. Gastrointestinal dysfunction is included as one of the multiple symptoms in ME/CFS. Although not required for a diagnosis, gastrointestinal abnormalities and comorbid irritable bowel syndrome are frequently reported by patients with ME/CFS [132]. ME/CFS patients more frequently experience gastrointestinal symptoms and use corresponding treatments (i.e., antacids, H2 blockers, proton pump inhibitors) compared with healthy controls [133]. Estimates based on a clinical patient group of 1400 patients show recurring gastrointestinal symptoms are experienced by between 80 and 90% of patients [134]. In a sample of 165 CFS patients, Chia and Chia [134] identified evidence of chronic inflammation and enterovirus of the stomach in 95 and 82% of patient biopsies respectively. As the authors suggest, the presence of viral markers in the stomach years after initial infection suggest that chronic viral infections of the stomach may contribute to continued pathophysiology. Viral infections have been proposed to precipitate and perpetuate the bacterial dysbiosis observed in ME/CFS (see review by Navaneetharaja et al. [135]).

### Bacterial dysbiosis, antibiotic and probiotic treatment

Evidence of gut dysbiosis has been observed through measurement of fecal microbial composition in ME/CFS populations. Differences between microbial composition of healthy compared with ME/CFS populations have been reported using both culture-based [136, 137] and genetic sequencing methods [3, 138]. Treatment using antibiotic [139], probiotic [140–142] or bacteriotherapy [143] have also been used to help modulate the gut microbiota in ME/CFS with somewhat unpredictable and varied success.

Using culture-based methods, we have previously observed a predominance of d-lactate producing bacteria (*Enterococcus* and *Streptococcus* species) in ME/CFS patients [4]. These bacteria produce high levels of lactate in vitro, compared with fecal isolates [4] which would support the maintenance of a more acidic colonic environment as one of the mechanisms in d-la that was previously described. This inference about the acidity of the colon in ME/CFS patients has been deduced from in vitro methods only, as we are not aware of any research that has measured colonic pH in this population. Within our prior clinical investigations, responders to a short-term antibiotic treatment for *Streptococcus* overgrowth was associated with increased vigor on self-reports and selected improvement on objective sleep markers [139]. Extending from these preliminary findings, we are currently examining interactions between microbiota, broader neuropsychological symptoms and d-lactate levels in a clinical pilot evaluating treatment aimed at reducing an overgrowth of *Streptococcus* in a subgroup of ME/CFS patients. Our group have also compared culture-based fecal assessment and symptom expression within a larger sample (*N* = 274) of ME/CFS patients [5]. This observational study showed partial support for d-lactate theory in ME/CFS whilst raising questions about sex differences. Significant positive associations between some lactate producing bacteria (*Lactobacillus* and *Streptococcus* genera) and ME/CFS symptoms were shown for males but not females [5, 144]. Notably, the relative abundance of genera measured was consistent across the sexes raising questions about the functional differences of microbiota or a differing response to d-lactate for males compared to females. The heterogeneity of presentation and differing response to treatments could have varied explanations. Through the d-lactate lens, a preferential uptake of d-lactate (i.e., d-lactate accumulation as proposed by Mayeur et al. [110]) may contribute to variable symptoms and/or treatment response.

Using sequencing methods, Frémont et al. [3] examined ME/CFS patients and healthy controls from Norway and Belgium. Comparison between patient and control groups revealed no significant difference in bacterial diversity across the groups but differences in composition were observed. When comparing Norwegian patient and control samples there was a significant difference in bacterial composition, with ME/CFS patients showing a lower proportion of genus within the *Firmicutes* phylum. Interestingly, microbial differences between culturally diverse control samples (i.e., Norwegian compared with Belgian; [3] highlight the importance of considering inter-individual characteristics that may contribute to microbial variation.

Unlike Frémont et al. [3] findings of similar bacterial diversity, Giloteaux et al. [138] reported evidence of decreased diversity of microbial composition and instability in the microbial community in ME/CFS patients compared with controls. Significant differences were not shown when comparing the composition of ME/CFS and control samples at the phylum or genus level. However, at the operational taxonomic unit (OTU) level, proportions significantly differed for 40 OTU’s. For example, the proportion of *Faecalibacterium* and *Bifidobacterium* was significantly lower in ME/CFS patients compared with controls. The few studies that have examined fecal microbial composition in ME/CFS have shown some inconsistent results making current interpretation incomplete suggesting that evaluation of subgroups, species-level comparison and measurement of metabolites is required. Replication using a combination of culturing and genetic sequencing methods with larger samples and varied demographics will help ascertain the relevance of d-lactate neurotoxicity in ME/CFS.

*Bifidobacterium* are high lactate-producing bacteria. Whilst the ratio of D/L lactate vary between species, a lower proportion of *Bifidobacterium* species raises some doubt about the relevance of d-lactate theory for ME/CFS. Selected *Bifidobacterium* (*Bifidobacterium adolescentis, Bifidobacterium breve*) and *Lactobacillus* (*L. plantarum, L. salivarius, L. casei subspecies rhamnosus, L. delbrueckii* subsp. *Lactis, L. acidophilus, L. fermentum, L. buchneri*) species have been identified as predominant in patients with d-la [26, 28, 29, 37, 47, 54, 64, 68, 74, 75, 145]. Similarity between species identified as overgrown in d-la patients and those used in probiotic studies could also generate skepticism about the relevance of d-lactate theory for ME/CFS. Both a small open-label [140] and two randomized, double-blind placebo-controlled studies [141, 142] examining the efficacy of probiotic supplementation in ME/CFS have indicated modest improvements for selected symptoms.

ME/CFS patients supplemented with a lactic-acid producing bacterial strain probiotic showed clinical improvement in self-reported neurological symptoms but no significant changes in fatigue or activity levels [140]. Rao et al.’s (2009) small double-blind RCT used an eight-week probiotic supplementation of *Lactobacillus casei* to examine changes in emotional symptoms. ME/CFS patients in the treatment group reported a significant reduction in anxiety symptoms compared with controls. No change was recorded for subjective reports of depression. More recently, treatment using *Bifidobacterium infantis* 35,624 resulted in reduced inflammation in ME/CFS patients, however neurological symptoms were not measured [142]. Preliminary results indicate the need for further investigation of the efficacy of probiotic treatment in ME/CFS. Of relevance to the current hypothesis in question, the d-lactate potential of selected strains used in the aforementioned studies is unknown. Therefore, results from these treatment studies suggest support for gut–brain interaction in ME/CFS but fail to provide additional information about the relevance of d-lactate for this population.

Bacterial overgrowth in the small intestine may also have implications for d-lactate production. Logan et al. [146] hypothesized that SIBO is involved in ME/CFS and related to the immune alterations observed in this condition. SIBO can be a cause of functional short bowel and result in carbohydrate malabsorption. Patients with comorbid SIBO and CFS have shown clinical improvement (on subjective reports of depression, memory/concentration and pain) following antibiotic treatment [101]. d-Lactate levels were not measured in this study but dependent on the type of bacterial overgrowth, excess production of bacterial metabolites (including but not limited to d-lactate) may act centrally, through ENS activation or systemically due to intestinal permeability.

### Implications for gut–brain interaction

Systemic inflammation as a consequence of gut mucosal damage and intestinal permeability as the first proposed pathway of gut–brain interaction in d-la has also been suggested as a pathophysiological mechanism in ME/CFS [116]. Initial support for this hypothesis in ME/CFS is reflected by findings of an increased immune response to lipopolysaccharide (LPS) (as measured by serum IgA and IgM to selected bacteria [116] and clinical improvement after treatment to restore intestinal permeability [113]. Measurement of plasma levels of LPS have been used as an indicator of microbial translocation as they are produced in response to Gram-negative bacteria [138]. Chronic LPS stimulation can be measured by plasma sCD14 and plasma LPS-binding protein (LBP) levels [138]. Recently, additional evidence of intestinal permeability in ME/CFS patients has been shown through significantly higher proportions of plasma LPS, LBP and sCD14 compared with controls [138]. These results support the hypothesis of an inflammatory and/or immune response to microbial translocation that occurs when there is chronic gut mucosal damage and intestinal permeability in ME/CFS patients.

### Nutritional deficiencies in ME/CFS

Nutritional status can be impaired for individuals with chronic health conditions and comorbid gastrointestinal abnormalities. Nutritional deficiencies require careful monitoring and treatment for ME/CFS patients [1]. Coenzyme Q10 (CoQ10) was shown to be significantly lower in the plasma of ME/CFS patients compared with healthy controls [147]. Treatment that includes nutritional supplementation is frequently employed with CoQ10, magnesium, l-carnitine and S-adenosylmethionine indicated as potentially beneficial for this population [148, 149]. Improvements in cognitive symptoms (mental fatigue, attention, concentration) have been described after supplementation with acetyl-L-carnitine and propionyl-L-carnitine for patients with ME/CFS [150]. Colabamin (B12) injections are proposed to exert effects by reducing oxidative stress [151] but the implications of B12 deficiency may also be relevant when considering the role of B12 in the TCA cycle and lactate metabolism (see [152]). Considering the impact of nutritional deficiencies in d-la, this may interact with the symptom presentation in ME/CFS and the potential for excess d-lactate accumulation or issues with metabolism. Nutritional deficiencies in ME/CFS may have varied origins, including but not limited to, genetic vulnerabilities, stress, infection, inadequate dietary sources and/or impaired metabolism that are factors involved in the etiology of ME/CFS [153]. Dietary modifications appear helpful for some ME/CFS patients (self-report in [154]) and in clinical case reports [148]. Similarly, dietary treatments and reduced carbohydrate intake were common recommendations for d-la patients (see Additional file 1: Table S1). It would be useful to understand the role of diet as a potential moderating factor (precedent, perpetuating or consequential) in bacterial dysbiosis and d-lactate production in ME/CFS patients and whether this varies for moderately impaired compared to severely impaired (i.e., bedbound) patients.

## Conclusions

d-la is an acute condition that provides a clear example of the microbe–gut–brain interaction with encephalopathy similar to ME/CFS. Growing evidence supports the proposal of the microbiota–gut–brain interaction in ME/CFS. Specific mechanisms are yet to be confirmed. Our qualitative review of d-la case studies shows considerable overlap between d-la and ME/CFS neurological symptoms. Subclinical levels of d-lactate may be related to fluctuating neurological symptoms in ME/CFS. Our review of the d-la literature has led us to propose the hypothesis that d-la and ME/CFS may lie on a continuum, with notable distinctions related to differences in acute versus chronic presentations (see Fig. 3). Increased prevalence of d-lactate producing bacteria in an ME/CFS sample compared with controls [4] provides the only clear evidence supporting d-lactate theory in ME/CFS. Gut dysbiosis in fecal microbiota, SIBO, and preliminary responses to antibiotics warrant measurement of d-lactate levels in this clinical population.

We acknowledge the complexity and heterogeneity of ME/CFS. Explanation of other pathophysiological mechanisms in ME/CFS (including but not limited to neuro-immune, oxidative stress and inflammatory pathways, [116, 147, 153, 155, 156] was beyond the scope of the current review. We stress that d-lactate theory may be relevant for a select subgroup and if not causative, may be a factor that perpetuates or exacerbates neurological symptoms. To date, there is no research that has measured d-lactate levels in ME/CFS. Improved efficiency and availability of d-lactate measurement in urine and blood samples is needed. Measurement of d-lactate will clarify its role of d-lactate in this population and may generate an avenue for alternative treatments. Subclinical levels of d-lactate in diverse populations suggest that this may be extended to other conditions. The proposed continuum is relevant for general physicians, gastroenterologists, psychiatrists and psychologists alike. Awareness of gastrointestinal origins for neurological presentations may hasten diagnostic accuracy, prevent misdiagnosis and improve treatment outcomes.

## Additional files

**Additional file 1: Table S1.** Demographic and clinical data summary of d-lactic acidosis episodes (*n* = 59) included in the qualitative synthesis. All episodes simultaneously reported at least one high d-lactate level (from blood or urine analysis) and documented neurological symptoms. Episodes were screened for information about patient demographics, neurological symptoms, non-neurological symptoms, d-lactate levels, l-lactate levels, anion gap, pH levels, microbial composition, proposed triggers, medical history/comorbid conditions and treatment. Numbers in brackets (1) and (2) indicate separate episodes for the same patient. The letters *a* and *b* identify different patient cases reported in the same reference. Episodes from non-SBS patients are marked with an asterisk.

**Additional file 2: Table S2.** Episodes that reported *matching* or *ambiguous/other* d-lactic acidosis (d-la) symptoms as a function of age and sex.

## Abbreviations

D-la: d-lactic acidosis
SBS: short bowel syndrome
ME/CFS: myalgic encephalomyelitis/chronic fatigue syndrome
d-2-HDH: d-2-hydroxy acid dehydrogenase
ENS: enteric nervous system
CNS: central nervous system
ATP: adenosine triphosphate
TCA: tricarboxylic acid
