# Supplementary material for: Examining clinical similarities between myalgic encephalomyelitis/chronic fatigue syndrome and d-lactic acidosis: a systematic review
Source: J Transl Med. 2017 Jun 7;15:129. doi: 10.1186/s12967-017-1229-1 (PMC5463382; doi:10.1186/s12967-017-1229-1)
Supplement: Supplementary file 2 — Additional file 2: Table S2. Episodes that reported matching or ambiguous/other d-lactic acidosis (d-la) symptoms as a function of age and sex. [file 12967_2017_1229_MOESM2_ESM.pdf]

Supplementary Information for

Examining Clinical Similarities between Myalgic Encephalomyelitis/Chronic Fatigue Syndrome and D-Lactic Acidosis: A Systematic Review

Amy Wallis[1], Michelle Ball[1], Sandra McKechnie[2], Henry Butt[3], Donald P Lewis[4] & Dorothy Bruck[1]

[1]Psychology Discipline, Victoria University, Victoria, Australia.
[2]College of Engineering & Science, Victoria University, Victoria, Australia.
[3]Bioscreen (Aust) Pty Ltd, Victoria, Australia.
[4]CFS Discovery Clinic, Donvale, Victoria, Australia.

This PDF file includes:

Supplementary Table 2. Episodes that reported *matching* or *ambiguous/other* D-lactic acidosis (D-la) symptoms as a function of age and sex.

Other supplementary material for this manuscript includes the following:

Additional file 1: Supplementary Table 1 (excel file) Demographic and clinical data summary of D-lactic acidosis episodes (*n* = 59) included in the qualitative synthesis.

Legend: All episodes simultaneously reported at least one high D-lactate level (from blood or urine analysis) and documented neurological symptoms. Episodes were screened for information about patient demographics, neurological symptoms, non-neurological symptoms, D-lactate levels, L-lactate levels, anion gap, pH levels, microbial composition, proposed triggers, medical history/comorbid conditions and treatment. Numbers in brackets (1) and (2) indicate separate episodes for the same patient. The letters *a* and *b* identify different patient cases reported in the same reference. Episodes from non-SBS patients are marked with an asterix (*).

Supplementary Table 2. Episodes that reported *matching* or *ambiguous/other* D-lactic acidosis (D-la) symptoms as a function of age and sex.

**ME/CFS ICC**	**D-la$_b$ symptom overlap with ME/CFS**	**Episode Case Numbers**				
		Paediatric (≤ 17 years)			Adult (≥ 18 years)	
		Male	*Female*	*NI*	*Male*	*Female*
**A.** Postexertional neuroimmune exhaustion	Matching	14, 48, 50, 66, 53, 8, 47a, 47b	40	-	36, 42	12b, 13, 58b, 39
	Ambiguous/other	-	-	-	-	-
**B.** Neurological impairments	Matching	48, 47a, 30, 19, 52, 56, 66, 43, 53, 14, 50, 8	37, 17, 51b, 4, 40, 35	11	12a, 10, 29, 44, 58a, 25, 24, 49, 64, 59, 25, 42, 62, 9, 15, 23, 27, 33	6, 16_1, 16_2, 65, 20, 34_2, 57, 28, 41, 58b, 60, 21, 12b, 13, 34_1
	Ambiguous/other	43, 56, 66, 14, 18, 51a, 8, 19, 48, 53, 30	35, 37	11	9, 29, 44, 58a, 42, 64, 15, 24, 25, 49, 55, 23, 26, 10, 62, 27, 33	57, 21, 13, 31, 39, 58b, 43_2, 6, 16_1, 16_2, 65, 20, 34_1, 12b
	Ambiguous/other B1-B4	43, 56, 66	35	11	9, 29, 44, 58a, 42, 64, 15, 24, 25, 49, 33	57, 21, 13, 31, 39, 58b
	Speech/Language	14, 48, 66, 53, 8, 30, 19	37, 35	11	10, 15, 44, 62, 64, 9, 23, 25, 58a, 27, 33	6, 16_1, 16_2, 65, 20, 34_1, 57, 21, 12b
	Consciousness	14, 18, 51a, 8, 19	-	-	55, 9, 23, 26, 49	34_2, 57, 21
**C.** Immune, gastrointestinal and genitourinary	Matching	50, 47b, 66	-	-	10, 62, 42, 44, 36, 15	16_2, 60, 16_1, 65
	Ambiguous/other	-	-	-	-	-

**D.** Energy production/ transportation impairments	Matching	56, 66, 48, 51a, 52, 8, 43, 19	51b, 17, 35	-	55, 42, 25, 49, 9, 29, 62	60, 39
	Ambiguous/other	-	-	-	42, 23	-
**Mood / Behavior**	Matching	52, 53	40	-	9, 10	60, 31, 16_2
	Ambiguous/other	50, 56, 30, 66, 53, 19	-	-	44, 58a, 62, 33	31, 58b, 20
**Uncategorized D-la Symptoms**						
	Metabolic acidosis	14, 18, 48, 50, 51a, 52, 66, 53, 56, 8, 43, 47a, 47b, 30, 19	35, 51b, 37, 40, 4, 17	11	10, 15, 29, 44, 55, 62, 64, 9, 12a, 23, 24, 25, 26, 49, 58a, 59, 27, 36, 42, 33	6, 12b, 16_1, 16_2, 60, 65, 13, 20, 31, 34_1, 34_2, 57, 58b, 21, 28, 41
	Other abnormalities	66, 43, 47a, 47b	-	-	9, 2, 15, 26, 59, 33	16_1, 58b

ambiguous/other: symptoms that were not clearly identified as consistent with ME/CFS presentation (see Table 2 for detailed symptom delineation); D-la: D-lactic acidosis; ICC: International Consensus Criteria; *matching*: mapped overlap between ME/CFS and D-la symptoms; ME/CFS: myalgic encephalomyelitis/chronic fatigue syndrome; NI: sex not identified.

Legend. Subscript numbers ($_1$ and $_2$) indicate separate episodes for the same patient. The letters *a* and *b* identify different patient cases reported in the same reference. ME/CFS broad category B. Neurological impairments are highlighted as the primary focus of this review and to show three subcategories of delineation under *ambiguous/other* symptoms (i.e., in accordance with specific ICC criteria (B1 – B4), speech/language symptoms, and level of consciousness; see Table 2). Therefore, the same episode code number can be shown several times to represent multiple symptoms during each D-la episode (see Table 1 for references). Descriptions of drunkenness were referred to in several studies. Adult males self-reported *feeling* “drunk” (12a, 27) whereas females were described as *appearing* “drunk” (16_1, 16_2, 20,

58b). Rather than using this ambiguous term, the specific symptoms that were also referred to in each of these studies were categorized in the table. See Table 3 for a summary of symptom frequencies.
